# Sleep Disorders in Pediatric Migraine: A Questionnaire-Based Study

**DOI:** 10.3390/jcm10163575

**Published:** 2021-08-14

**Authors:** Alessandra Voci, Oliviero Bruni, Michela Ada Noris Ferilli, Laura Papetti, Samuela Tarantino, Fabiana Ursitti, Giorgia Sforza, Federico Vigevano, Luigi Mazzone, Massimiliano Valeriani, Romina Moavero

**Affiliations:** 1 Child Neurology and Psychiatry Unit, Systems Medicine Department, Tor Vergata University of Rome, 00133 Rome, Italy; Alessandra.voci@gmail.com (A.V.); luigi.mazzone@uniroma2.it (L.M.); 2 Department of Developmental and Social Psychology, Sapienza University, 00185 Rome, Italy; Oliviero.bruni@uniroma1.it; 3 Headache Center, Child Neurology Unit, Neuroscience Department, Bambino Gesù Children’s Hospital, IRCCS, 00165 Rome, Italy; michela.ferilli@opbg.net (M.A.N.F.); laura.papetti@opbg.net (L.P.); samuela.tarantino@opbg.net (S.T.); Fabiana.ursitti@opbg.net (F.U.); Giorgia.sforza@opbg.net (G.S.); federico.vigevano@opbg.net (F.V.); Massimiliano.valeriani@opbg.net (M.V.); 4 Center for Sensory Motor Interaction, Aalborg University, 9220 Aalborg, Denmark

**Keywords:** sleep, sleep disorders, headache, migraine, CSHQ

## Abstract

There is a high comorbidity between migraine and sleep disorders (SD), with a mutual dependence between sleep and headache. This study aimed to analyze the relationship between headache features (migraine frequency and severity, migraine equivalents, use and efficacy of treatments) and sleep in pediatric migraine. Parents of children and adolescents with migraine completed the Children’s Sleep Habits Questionnaire (CSHQ) and the Epworth Sleepiness Scale for Children and Adolescents (ESS-CHAD) and answered questions about headache characteristics. The presence of SD was defined according to CSHQ score. SD were detected in 72.9% of 140 subjects, but only 5.0% already received a diagnosis. Patients with SD presented statistically significant higher headache frequency (*p* = 0.031) and higher prevalence of migraine equivalents (*p* = 0.007). A higher CSHQ total score was associated with higher frequency of severe attacks (*p* = 0.012) and lower acute drug efficacy (*p* = 0.003). Significant positive correlations of sleep onset delay, sleep duration and nightwakings subscales with migraine frequency emerged. Our findings indicate that SD are highly prevalent in pediatric migraine and frequently associated with a higher headache severity and lower response to acute therapy, but often remain underdiagnosed. Improving sleep quality could help to reduce migraine intensity and disability and vice versa.

## 1. Introduction

Sleep disorders (SD) are very common in pediatric age and about 25% of children experience a sleep problem at some point before adulthood, with higher rates if other medical, psychiatric, neurodevelopmental, and neurologic conditions exist [1,2]. Among neurologic disturbances, migraine has a strong and complex relationship with SD, although the precise nature of this association is still enigmatic [3].

There is a mutual dependence between sleep and headache. Inadequate or prolonged sleep duration or poor sleep quality are triggering factors for migraine attacks, and frequent nocturnal awakenings precede sleep-related migraine [4,5]. Furthermore, bad sleep has been related to migraine chronicization [6].

On the other hand, migraine can be seen as a possible cause of sleep alteration: indeed, having a nocturnal attack may disrupt sleep [4]. Moreover, many children with migraine tend to fall asleep, as a therapeutic method to terminate pain: this could lead to disruption of the normal sleep-wake cycle [7].

Children with migraine might also experience excessive daytime sleepiness [6,8,9], although some authors hypothesized that this might be due to other mood or medical disorders, poor sleep quality, medications, and greater headache intensity and disability [6,10].

The high comorbidity rate between migraine and SD might be partially explained by the alteration of the serotoninergic system, which plays a role both in nociception and in sleep regulation [11,12]. In addition, the presence of common risk factors such as mood and anxiety disorders, related to both migraine and sleep disturbances, might play a role [13].

Furthermore, migraine and SD could represent an expression of a common pathogenic process [3,5,14]. In recent years, increasing scientific evidence supported this theory and the presence of common cerebral structures and signaling pathways (such as the hypothalamus, raphe nuclei and serotoninergic system), involved in both sleep and migraine, has been underlined [4].

Finally, both SD and migraine have been linked to vitamin D deficiency [15,16,17], therefore this can act as a common predisposing factor. In specific conditions, vitamin D supplementation might have an important role in the treatment of both disorders [17,18].

SD and migraine are also considered to be part of a cluster of recurrent clinical symptoms included in the so-called “episodic syndromes that may be associated with migraine” (also named “migraine equivalents or migraine variants”) [5]. These conditions are considered early life expressions of migraine [19], with a periodic or paroxysmal pattern. The new ICHD-3 classification [20] includes in this spectrum of disorders recurrent abdominal pain, cyclical vomiting syndrome, abdominal migraine, benign paroxysmal vertigo, benign paroxysmal torticollis, infantile colic, alternating hemiplegia of childhood, and vestibular migraine. Additional conditions included are motion sickness and specific sleep disorders (such as sleep walking, sleep talking, night terrors and bruxism) [21].

In the last three decades, a few studies examined patients with headache through the administration of standardized questionnaires for the evaluation of SD [5,8,14,22]. Parental reports of SD are consistent with objective measurements such as actigraphy and polysomnography [22]. According to these studies, pediatric patients with migraine appeared to be at increased risk of developing SD compared to those with tension-type headache, presenting a variety of disorders including poor sleep quality, night awakenings, nocturnal symptoms and daytime sleepiness [22]; furthermore, an association between SD and migraine-related disability and intensity has been proposed [5]. However, most of these studies were not specifically designed for migraine, including all primary headaches.

To the best of our knowledge there are no reports on the prevalence of migraine equivalents in children with and without SD. Considering the emerging thesis that SD could be part of this spectrum of disorders related to migraine [23,24], a better evaluation of a possible co-occurrence of the classical and yet established migraine equivalents with SD is mandatory.

Primary aim of the present study was to analyze the prevalence of SD in children and adolescents with migraine through the use of two structured and validated questionnaires (Children’s Sleep Habits Questionnaire (CSHQ) and Epworth Sleepiness Scale for Children and Adolescents (ESS-CHAD)). Secondary aims were: (1) to evaluate differences between migraine pattern, frequency and intensity, and the use of a therapy for attacks or prophylactic medications in subjects with and without SD; (2) to investigate the prevalence of migraine equivalents and find an eventual correlation with SD; (3) to evaluate the possible influence of concurrent conditions such as developmental or medical diseases, anxiety disorders, and chronic pharmacological therapy.

## 2. Materials and Methods

### 2.1. Subjects Recruitment

We identified potential subjects for our study among consecutive patients from the Pediatric Headache Center of the Bambino Gesù Children’s Hospital in Rome.

We included all patients fulfilling the following criteria:

1. Migraine (with or without aura) diagnosis according to the ICHD-3 criteria [20];
2. Maximum age of 18 years;
3. A headache history of at least one year;
4. At least one follow-up examination at the Bambino Gesù Pediatric Hospital in the previous year.

Exclusion criteria included a diagnosis of tension-type headache alone and the inability to complete the questionnaires.

### 2.2. Questionnaire Structure and Procedures

To investigate the presence of SD in pediatric patients with migraine, we created an online questionnaire using the “Google Forms” tool provided by Google. We asked parents to fill out the questionnaire by following a link sent by e-mail.

The questionnaire was structured as follows:

1. A first part with general questions on clinical characteristics of migraine in the child (e.g., diagnosis of migraine with/without aura, localization and quality of pain, associated symptoms, eventual aura manifestations, frequency and intensity of attacks, type and dosage of drugs used for migraine attacks and their effectiveness in the previous two months, prophylaxis therapies and their efficacy) (supplementary document 1);
2. Questions related to the presence of migraine equivalents in the history of the child;
3. A last section containing the two standardized questionnaires CSHQ and ESS-CHAD (full version for both questionnaires).

### 2.3. Sleep Evaluation

#### 2.3.1. Children’s Sleep Habits Questionnaire

The CSHQ is one of the most used assessment tools for pediatric sleep [25]. It is a parent-report sleep screening survey, specifically designed for school-aged children, and later extended for adolescents [26]. The questionnaire has shown adequate reliability and validity [5,26].

The questionnaire consists of 33 items grouped in 8 subscales relating to the major presenting clinical sleep complaints in pediatric age: bedtime resistance, sleep onset delay, sleep duration, sleep anxiety, night wakings, parasomnias, sleep-disordered breathing, and daytime sleepiness. Each item is rated on a three-point scale, and items questioning about desirable and pathological sleep behaviors are reversed in scoring so that a higher score is indicative of more disrupted sleep.

According to the CSHQ total score (cut-off 41) [26], patients were categorized as with or without SD.

#### 2.3.2. Epworth Sleepiness Scale for Children and Adolescents

The ESS-CHAD has been proposed as the modified version for children and adolescents of the Epworth Sleepiness Scale (ESS), firstly developed as a measure of daytime sleepiness in adults [27]. The ESS-CHAD contains eight questions in which the patient or their caregiver is asked to rate the child’s usual chances of falling asleep while doing different activities. The total score ranges from 0 to 24, with higher scores representing greater sleepiness. A score above 10 suggests excessive daytime sleepiness [28].

### 2.4. Statistical Analysis

Statistical analysis was performed using Statistical Package for the Social Sciences (IBM SPSS Statistics version 22.0). We used chi-square and F tests to compare headache features between patients with and without SD. For numeric variables (e.g., age at onset of migraine), we compared the two groups using a *t*-test. Uni- and multivariate logistic regression models with “SD” and “no SD” as dichotomous outcome variables were used to estimate the odds ratios (OR) of several possible explanatory covariates (such as other chronic medical conditions and pharmacotherapies).

Spearman’s rank coefficient was used to correlate ordinal categorical variables related to headache characteristics (habitual and current frequency of attacks, occurrence of severe attacks, number of acute drug medications and their efficacy, expressed in ranges) with CSHQ scores (total, subscales’ and specific items’ scores). Pearson’s correlation was performed to analyze the relationship between numeric variables.

*p* values < 0.05 were considered statistically significant.

## 3. Results

We collected a total of 143 questionnaires; 3 were excluded (tension-type headache or older than 18 years). Therefore, 140 questionnaires underwent our final analysis.

Of the 140 children and adolescents participating in the study, 54 were males (38.6%) and 86 females (61.4%), with an age range of 3–18 years (mean 12.1 ± 3.6 years, median 12.4 years). Migraine without aura (MoA) was present in 125 patients (89.3%), while 15 (10.7%) had migraine with aura (MwA). Ten patients (7.1%) fulfilled the criteria for chronic migraine (CM), all with MoA. Mean age at migraine onset was 7.4 ± 3.6 years (median 7.5 years).

Only 7/140 patients (5.0%) already received a previous diagnosis of SD (sleep onset delay and night wakings); four of them reported previous treatment with melatonin or tryptophan, and none underwent specific sleep evaluation in the past.

One-hundred and two subjects (72.9%) had a CSHQ total score above the cut-off, and thus were diagnosed with a SD.

**Figure 1:**
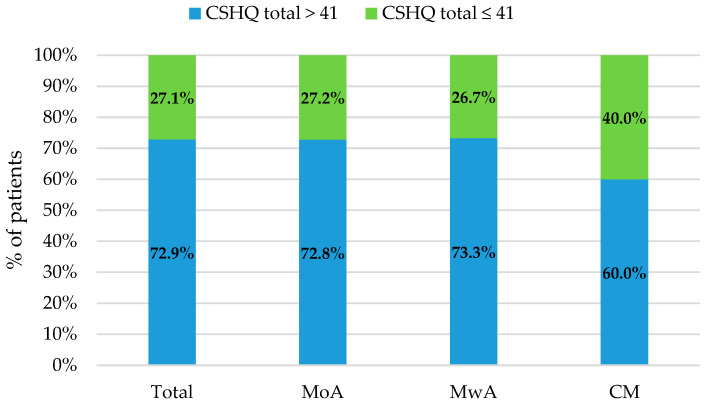
Percentage of patients with total CSHQ score above and below the cut-off considering the whole group, and the subgroups of MoA, MwA and CM.

The ratios of subjects who scored above and below the cut-off in CSHQ (taking into account the whole group and MoA, MwA and CM subgroups) are reported in Figure 1.

Patients with a total score above the cut-off in CSHQ were 91/125 (72.8%) among subjects with MoA, 11/15 (73.3%) considering MwA, and 6/10 (60.0%) for CM.

We compared the clinical and migraine characteristics of patients with and without SD. Results are summarized in Table 1.

Concerning the frequency of attacks reported by each patient in the two months prior to the completion of the questionnaire, 20 patients (14.3%) reported no episodes, 62 (44.3%) reported 1–3 attacks per month, 40 (28.6%) reported 4–8 episodes per month, 14 (10.0%) reported 9–14 attacks, and 4 (2.9%) reported 15 or more attacks per month. Children with SD were more likely to present more than eight episodes per month when compared to children without (17.5% vs. 10.5%, *p* = 0.031).

Considering the specific symptoms accompanying headache, we found a statistically significant higher prevalence of vertigo in subjects with SD compared to those without (39.2% vs. 18.4%, *p* = 0.021). No statistically significant differences were found for phonophobia, photophobia, nausea, and vomiting.

Migraine equivalents were reported by 82.4% of patients with a SD versus 60.5% of patients without (*p* = 0.007). Considering individually the different manifestations, a statistically significant difference was found only for benign paroxysmal vertigo (*p* = 0.047) and cyclic vomiting syndrome (*p* = 0.016).

Almost all (139/140, 99.3%) of our patients used symptomatic drugs in case of migraine attack; 97.8% of these patients used acetaminophen and/or non-steroidal anti-inflammatory drugs and 2.2% used triptans. Thirty-one patients (22.1%) did not need any attack medications in the previous two months, 62 patients (44.3%) took medications 1–3 times, 37 patients (26.4%) took symptomatic drugs for 4–8 attacks, 9 (6.4%) reported 9–14 drug assumptions, while only one patient took 15 or more drugs in the previous two months. No differences in distributions between children with and without SD were observed.

Considering the efficacy of the symptomatic therapy, 56 children with SD (54.9%) reported a good efficacy, while partial or no efficacy were reported by 40 (39.2%) and 6 (5.9%) patients, respectively. In children without a SD, 29 (76.3%), 9 (20.0%) and no patients reported respectively good, partial and no efficacy (*p* = 0.045).

Twenty-one subjects in the study (15.0%) were on prophylactic treatment. Drugs included amitriptyline (14.3%), flunarizine (47.6%), topiramate (23.8%), tryptophan (4.7%), and palmitoylethanolamide (19.0%); one patient was taking cyproheptadine to prevent cyclic vomiting, while another one was on the ketogenic diet. No differences were found in distribution of SD between patients with ongoing prophylaxis and those without.

Taking into account the co-occurrence of various comorbidities (as reported by parents in the questionnaire), we found that 4 patients (2.8%) had a history of psychomotor delay, 7 (5.0%) had a history of language delay, 3 patients (2.1%) had a diagnosis of intellectual disability, 4 patients (2.8%) had a diagnosis of of Attention Deficit–Hyperactivity Disorder (ADHD), 11 patients (7.8%) reported academic difficulties, while 30 patients (21.4%) suffered from anxiety and 5 (3.6%) from mood disorder. Twenty-six cases (18.6%) had other chronic medical conditions (allergy, asthma, mild heart diseases, endocrine pathologies, celiac disease, gastroesophageal reflux, epilepsy). Regular medications for chronic diseases were being used by 17 patients (12.1%): these included vitamin supplements, inhaled bronchodilators, oral antihistamines, anti-hypertensive drugs, levothyroxine, triptorelin, desmopressin, and levetiracetam, while two patients were taking melatonin and tryptophan for sleep. We found a higher prevalence of SD in children with neuropsychiatric comorbidities (*p* = 0.026). This association was also confirmed by a univariate logistic regression analysis, which estimated that a neuropsychiatric comorbidity increases the risk of SD in migraine patients (*p* = 0.03, OR = 1.01). In particular, a correlation with anxiety and mood disorder emerged (*p* = 0.016). After adding in a multivariate logistic model, the independent variables of other chronic diseases and other pharmacotherapies, the presence of a neuropsychiatric condition still continued to be a significant risk factor for positive CSHQ (*p* = 0.04, OR = 0.95).

Spearman’s rank test was used to describe correlations between migraine and sleep characteristics (Table 2).

Total sleep time was significantly negatively correlated with the habitual frequency of the attacks before the completion of the questionnaire (r = −0.29, *p* < 0.001 and r = −0.17, *p* = 0.048).

A higher CSHQ total score was related to a higher frequency of severe attacks (r = 0.21, *p* = 0.012) and lower acute drug efficacy (r = −0.25, *p* = 0.003).

We also found statistically significant correlations of some CSHQ subscales with migraine characteristics (Table 2). “Sleep onset delay” and “Sleep duration” scores positively correlated with the habitual and current frequency of attacks and the intake of acute medications. A higher “Sleep duration” subscale’s score (meaning decreased sleep duration) was also associated with a higher severity of migraine attacks, while the “night wakings” subscale positively correlated with current frequency of attacks and occurrence of severe attacks, and negatively correlated with acute drug efficacy.

In order to understand if there are differences in SD between younger children and adolescents, Pearson’s correlation was performed to analyze the relationship between our patients’ age and CSHQ total and subscales’ scores. The “Sleep duration” subscale score significantly correlated with age, expressed in years (r = 0.34, *p* < 0.001). In addition, we found statistically significant negative correlations between age and “Bedtime resistance” and “Sleep anxiety” subscales’ scores (r = −0.41, *p* < 0.001 and r = −0.40, *p* < 0.001). No statistically significant correlation with the total CSHQ score emerged.

Only 4 patients (2.8%) reached a clinically significant score in the ESS-CHAD. None of these patients were under prophylactic therapy, while one patient was taking chronic pharmacological therapy with an antihistamine (cetirizine). Two of these patients also had clinically significant scores in the CSHQ “Night Wakings” subscale.

## 4. Discussion

Our study demonstrated that 72.9% of our population experience SD, underlining their relevance in migraine. Our results are consistent with prior pediatric questionnaire-based research reporting different types of SD in subjects with migraine and increased rates if compared to subjects with non-migraine headaches or healthy children [5,9,13,22].

Differently from a previous study, our research highlighted the correlation between SD and migraine frequency, intensity, and prophylactic treatment, but also evaluated the correlation with migraine equivalents and comorbidities of migraine.

A clinically relevant finding of the present study is that SD in pediatric patients with migraine may remain underdiagnosed in many cases: in fact, only 5.0% of our patients had already received a diagnosis of SD, and none of them had undergone specific evaluation. Although the precise reasons for this remain unknown, it is possible that patients with migraine attribute all their disturbances to migraine itself, thus not searching for specific help in the case of sleep disruption. This demonstrates the importance of performing a careful analysis of sleep habits during the clinical evaluation of patients with migraine, in order to identify the presence of possible sleep problems. A prompt and precise identification of this comorbidity can also help in finding the best treatment option; melatonin for instance can significantly reduce sleep onset latency but also modulates cytokines, interleukins, and TNF-alpha, thus playing a role in pain reduction [29,30]. For these reasons, melatonin is sometimes used in migraine prophylaxis, with greater effects when higher doses (3 mg) are used [31]. A possible use as an acute therapy has also been proposed, with the evidence of some benefits but a high drop-out from the study [32].

In our sample, children with SD also presented with a worse response to abortive treatment for migraine attacks. Disturbed sleep can be associated with higher nociceptive perception due to increased central sensitization and impairment of descending pain inhibitory capacity [33,34]. Furthermore, several animal models show that sleep deprivation is associated with altered endorphins in the plasma and hypothalamus [35], with a reduced sensitivity to opioid analgesics [36]. However, there are no reports on the mechanisms underlying a reduced efficacy of other analgesic treatments in the case of SD.

Our findings showed no differences in the presence of SD between patients with ongoing prophylaxis and those without, in disagreement with a previous report showing that migraine patients taking prophylactic medication reported significantly better total sleep scores. However, this discordant result could also be attributed to the low number of patients under prophylaxis in our sample.

The higher rate of SD in patients experiencing vertigo during migraine episodes could be due to the higher disability of migraine attacks with vertigo, with the need for interrupting activities, rest, and eventually falling asleep likely favoring the alteration of sleep-wake cycles [6,10,37], although this remains a mere hypothesis.

In our sample, we demonstrated a significant association between episodic syndromes and SD, consistent with the idea that SD could be considered part of a spectrum of disorders related to migraine or, as suggested, of child migraine syndrome [23,24]. An analysis of specific migraine equivalents revealed a significant association of benign paroxysmal vertigo and cyclic vomiting syndrome with SD. However, the lack of significant association with the other specific episodic syndromes could be partially influenced by the scarce numerosity of each subgroup.

Although some medications can have adverse consequences on sleep, in our sample, no differences emerged comparing subjects under chronic pharmacological therapy for systemic comorbidities with those without.

We found that decreased total sleep time, as well as longer sleep onset delay and shorter sleep duration, were associated with higher frequency of migraine episodes, in line with previous studies suggesting an association between greater sleep problems and higher headache severity [5,14]. Furthermore, children with a worse sleep quality are more prone to developing severe migraine attacks and to have lower efficacy in the use of abortive treatment.

Although we are not able to determine whether SD promotes migraine attacks or, conversely, migraine causes SD, we can state that the higher the severity of migraine, the more significant the alterations of sleep, and vice versa, and this can also be applied to the efficacy of therapy. Thus, investigation and treatment of comorbid SD and sleep hygiene interventions should always be considered in the management of migraine patients, since an improvement in sleep is expected to determine a reduction of headache severity and disability [38].

In our sample, a higher percentage of patients with SD presented with anxiety and mood disorders if compared to patients without sleep alterations, confirming previous findings [39]. Indeed, anxiety is a well-known comorbidity in both headache and sleep disorders [40,41]. In particular, a reciprocal relationship exists between anxiety and sleep quality, since anxiety can determine the difficulty in resting and falling asleep, thus determining a higher risk of insomnia and poor sleep quality [41,42]. On the other hand, REM sleep plays a key role in contributing to emotion regulation capacities, therefore REM sleep deprivation might significantly contribute to emotional disturbances, including anxiety disorder [41,43]. A survey suggested that the association between headache and sleep problems could be partially attributable to comorbid mood and anxiety disorders [13]. Further analysis is needed to verify and understand the degree of this association.

Concerning the distribution of SD between ages, no differences in CSHQ total score, and therefore in the presence of SD, were found in our sample. However, among SD, an inadequate sleep duration appears higher in older patients, while bedtime resistance tends to increase with decreasing age. In addition, sleep anxiety is more frequent in younger children, consistent with the present literature reporting that nighttime fears are quite common in young children [44].

Despite the high rate of SD, very few patients in our sample had pathological scores at ESS-CHAD, and no correlation of “Daytime Sleepiness” subscale of CSHQ with migraine severity emerged. Thus, in contrast to previous research [8], excessive somnolence is not among the major complaints of our sample of children with migraine. The presence of daytime sleepiness, reported by some patients, might be better explained as a sensation of fatigue during the day or could be secondary to other SDs, such as insomnia, that rarely have a ESS > 10 [6].

Our study has some limitations. First, data collection was based on parental reports via questionnaires, with a lack of objective sleep measures and only a retrospective analysis of patients’ sleep habits. Second, subjects were recruited from a tertiary center, which might have selected more severe cases of migraine. Third, a possible partial influence of a selection bias on our results must be considered: parents of children with more sleep difficulties, and therefore sensitive to the topic, may have answered the questionnaire. Fourth, the design of our research lacked a control group and did not allow us to determine the nature and the direction of the association between SD and migraine. Future work based on longitudinal analysis of migraine characteristics and sleep behaviors and alterations, with the use of daily reports or objective sleep measures such as polysomnography or actigraphy, would help to better characterize their relationship.

## 5. Conclusions

Our findings indicate that SD, though highly prevalent in pediatric migraine and frequently associated with a higher headache severity, remains underdiagnosed in many cases. Given the relationship between sleep and migraine characteristics, improving sleep quality could help to reduce migraine disability and vice versa. Therefore, the clinical evaluation of pediatric patients with migraine should always include a careful analysis of their sleep habits in order to detect the presence of SD early. Sleep hygiene interventions and treatment of SD could improve the frequency and the intensity of migraine attacks. Moreover, implementing migraine treatment strategies, with the aid of both pharmacological and behavioral therapies, could potentially prevent patient’s sleep disruption.

## Figures and Tables

**Table 1:** Comparison between migraine patients with and without sleep disorder according to CSHQ’s and SDSC’s scores.

Feature	Total	Sleep Disorder	Non-Sleep Disorder	*p*
	Mean ± SD; Median	Mean ± SD; Median	Mean ± SD; Median	
Age in years	12.1 ± 3.6; 12.4	11.7 ± 3.6; 12.1	13.1 ± 3.4; 13.3	0.033
Age at migraine onset in years	7.4 ± 3.6; 7.5	7.1 ± 3.7; 7.2	8.3 ± 3.4; 8.0	NS
Sex	% (Number)	% (Number)	% (Number)	
Males	38.6 (54)	39.2 (40)	36.8 (14)	
Females	61.4 (86)	60.8 (62)	63.2 (24)	NS
Type of migraine				
Migraine without aura	89.3 (125)	89.2 (91)	89.5 (34)	
Migraine with aura	10.7 (15)	10.8 (11)	10.5 (4)	NS
Episodic migraine	92.9 (130)	94.1 (96)	89.5 (34)	
Chronic migraine	7.1 (10)	5.9 (6)	10.5 (4)	NS
Manifestations of migraine				
Phonophobia	65.0 (91)	66.7 (68)	60.5 (23)	NS
Photophobia	67.1 (94)	67.6 (69)	65.8 (25)	NS
Nausea	49.3 (69)	51.0 (52)	44.7 (17)	NS
Vomiting	26.4 (37)	22.5 (23)	36.8 (14)	NS
Vertigo	33.6 (47)	39.2 (40)	18.4 (7)	0.021
Migraine equivalents	76.4 (107)	82.4 (84)	60.5 (23)	0.007
Infantile colic	35.0 (49)	33.3 (34)	39.5 (15)	NS
Recurrent gastrointestinal pain	26.4 (37)	29.4 (30)	18.4 (7)	NS
Benign paroxysmal vertigo	18.6 (26)	22.5 (23)	7.9 (3)	0.047
Cyclic vomiting syndrome	14.3 (20)	18.6 (19)	2.6 (1)	0.016
Recurrent limb pain	32.1 (45)	34.3 (35)	26.3 (10)	NS
Motion sickness	32.9 (46)	33.3 (34)	31.6 (12)	NS
Benign paroxysmal torticollis	3.6 (5)	3.9 (4)	2.6 (1)	NS
Familiarity for migraine	90.0 (126)	90.1 (92)	89.5 (34)	NS
Habitual frequency of attacks				
≤2 attacks/week	80.0 (112)	78.4 (80)	84.2 (32)	
>2 attacks/week	20.0 (28)	21.6 (22)	15.8 (6)	NS
Frequency of attacks in the last 2 months				
≤2 attacks/week	72.8 (102)	86.3 (88)	89.5 (34)	
>2 attacks/week	12.9 (18)	17.5 (14)	10.5 (4)	0.031
Severe attacks in the last 2 months				
≤2 attacks/week	96.4 (135)	95.1 (97)	100.0 (38)	
>2 attacks/week	3.6 (5)	4.9 (5)	0.0 (0)	NS
Symptomatic drug intake in the last 2 months				
≤2 medications/week	92.9 (130)	91.2 (93)	97.4 (37)	
>2 medications/week	7.1 (10)	8.8 (9)	2.6 (1)	NS
Efficacy of current acute medications				
Effective	60.7 (85)	54.9 (56)	76.3 (29)	
Partially effective	35.0 (49)	39.2 (40)	23.7 (9)	
Ineffective	4.3 (6)	5.9 (6)	0.0 (0)	0.045
Ongoing prophylactic treatments	15.0 (21)	17.6 (18)	7.9 (3)	NS
Neuropsychiatric comorbidities	32.9 (46)	38.2 (39)	18.4 (7)	0.026
Anxiety or mood disorders	25.0 (35)	30.4 (31)	10.5 (4)	0.016
Systemic comorbidities	18.6 (26)	19.6 (20)	15.8 (6)	NS
Chronic pharmacological therapy	13.6 (19)	16.7 (17)	5.3 (2)	NS

Abbreviations: NS: Non significant; SD: Standard Deviation.

**Table 2:** Relationship between migraine and sleep characteristics.

	Habitual Frequency of Attacks		Current Frequency of Attacks		Severe Attacks		Acute Drug Intake		Acute Drug Efficacy	
	r	*p*	r	*p*	r	*p*	r	*p*	r	*p*
Total sleep time	**−0.29**	**<0.001**	**−0.17**	**0.048**	−0.10	NS	−0.20	NS	0.13	NS
CSHQ total	0.12	NS	0.16	NS	**0.21**	**0.012**	0.15	NS	**−0.25**	**0.003**
CSHQ subscales										
BR	0.00	NS	0.06	NS	0.00	NS	0.06	NS	−0.10	NS
SOD	**0.24**	**0.004**	**0.23**	**0.006**	0.15	NS	**0.22**	**0.009**	−0.11	NS
SD	**0.17**	**0.044**	**0.24**	**0.005**	**0.22**	**0.009**	**0.21**	**0.014**	−0.10	NS
SA	0.05	NS	0.06	NS	0.12	NS	0.03	NS	0.17	NS
NW	0.10	NS	**0.17**	**0.047**	**0.27**	**0.001**	0.14	NS	**−0.22**	**0.008**
*p*	0.00	NS	0.02	NS	0.11	NS	0.05	NS	−0.15	NS
SDB	0.05	NS	0.07	NS	0.04	NS	0.09	NS	−0.09	NS
DS	0.04	NS	0.03	NS	0.08	NS	0.00	NS	−0.11	NS

Abbreviations: r: Spearman’s rank correlation coefficient; NS: Non significant; BR: Bedtime Resistance, SOD: Sleep Onset Delay, SD: Sleep Duration, SA: Sleep Anxiety, NW: Night Wakings, *p*: Parasomnias, SDB: Sleep Disordered Breathing, DS: Daytime Sleepiness. Significant results are highlighted in bold.

## Supplementary Materials

The following are available online at https://www.mdpi.com/article/10.3390/jcm10163575/s1, Document 1: questions related to headache features.
