# Supplementary material for: Sleep Disorders in Pediatric Migraine: A Questionnaire-Based Study
Source: J Clin Med. 2021 Aug 14;10(16):3575. doi: 10.3390/jcm10163575 (PMC8396839; doi:10.3390/jcm10163575)
Supplement: Supplementary file 1 [file jcm-10-03575-s001.zip › jcm-1321284-supplementary.pdf]

- Age at headache onset
- Did you child receive a specific diagnosis?
    - Migraine with aura
    - Migraine without aura
    - Tension type headache
    - He doesn't have a diagnosis
    - Other (specify)
- Which is the pain localization during headache attacks?
- Mono- or bilateral pain?
- Could you define the pain as gravative or pulsating?
- Is the pain accompanied by other manifestations?
    - Photofobia
    - Phonofobia
    - Nausea
    - Vomiting
    - Vertigo
    - None
    - Other(specify)
- Is there any aura?
- Did your child present any of the followings?
    - Infantile colic
    - Recurrent abdominal pain
    - Vertigo
    - Cyclical vomiting
    - Limb pain
    - Kinetosis
    - Paroxysmal torticollis
- Is there a positive familial history for headache?
- Which is the habitual frequency of headache attacks?
- Which was the frequency of attacks in the last 2 months?
- How many times did the headache cause the child interrupting his daily activities?
- How many times did your child need an abortive medication for headache in the last 2 months?
- Which drugs did he usually take in case of headache?
- Are these drugs usually effective?
- Did he take, or is he actually taking, any prophylaxis treatment? And which?
- Are they effective?
